# Knowledge, attitudes and perceptions about rabies among the people in the community, healthcare professionals and veterinary practitioners in Bangladesh

**DOI:** 10.1016/j.onehlt.2021.100308

**Published:** 2021-08-14

**Authors:** Md Sohel Rana, Afsana Akter Jahan, S.M. Golam Kaisar, Umme Ruman Siddiqi, Subir Sarker, Mst Ismat Ara Begum, Sumon Ghosh, Sudeb Sarker, Be-Nazir Ahmed, Abul Khair Mohammad Shamsuzzaman

**Affiliations:** a Communicable Disease Control (CDC), Directorate General of Health Services, Ministry of Health and Family Welfare, Dhaka 1212, Bangladesh; b Department of Veterinary and Animal Sciences, University of Rajshahi, Rajshahi 6205, Bangladesh; c International Centre for Diarrhoeal Disease Research, Bangladesh (ICDDR,B), Dhaka 1212, Bangladesh; d Department of Livestock Services, Ministry of Fisheries and Livestock, Dhaka 1215, Bangladesh; e School of Environmental and Rural Science, University of New England, Armidale, NSW 2351, Australia; f Department of Physiology, Anatomy and Microbiology, School of Life Sciences, La Trobe University, Melbourne, VIC 3086, Australia

**Keywords:** Attitudes, Bangladesh, Dog bite, Knowledge, Perception, Rabies, Zoonotic

## Abstract

It is crucial to explore knowledge, attitudes and perceptions (KAP) about rabies among the people in the community, the personnel dealing with animal bite management and suspected rabies patients, including humans and animals, to facilitate intervention in improving rabies elimination strategies. In 2016, we conducted an interactive face-to-face survey in three different districts of Bangladesh to understand the extent of KAP towards rabies in the community peoples (CPs), human healthcare professionals (HCPs) and veterinary practitioners (VPs). A set of prescribed questions was employed to measure what proportion of each group possessed sufficient knowledge, positive attitudes and adequate perceptions about rabies. A total of 1133 CPs, 211 HCPs and 168 VPs were interviewed by using a standard questionnaire comprising both closed and open-ended questions. Of the CPs, 49% identified the disease correctly (i.e. rabies is caused by an animal bite or a scratch). Only 29% of the CPs were aware that a wound should be washed immediately with soap and water after an animal bite or a scratch. However, only 49% of the CPs, 65% of the HCPs and 60% of the VPs felt that it is important to consult a physician and receive post-exposure vaccine as the first line of treatment following an animal exposure. Among the HCPs, 23% of the respondents did not possess sufficient knowledge about animal bites as categorised by the World Health Organization (WHO), and 12% of the respondents did not possess the knowledge on how to manage an animal bite properly. Out of 52% of the VPs who previously treated suspected rabid animals, only 29% had a history of taking rabies pre-exposure prophylaxis (PEP). Lack of formal education and rural subsistence were found to largely contribute to poor rabies KAP level among the CPs (*P* ≤ 0.01). There has been a high demand for proper training to be provided to HCPs and VPs for the effective management of an animal bite incidence in human and animals, respectively. Multi-sectoral collaboration through integrated One Health initiatives including community education, awareness programmes, facilitation of rabies PEP, and dog vaccination as well as its population control are critical in the way forward to control rabies in Bangladesh.

## 1 Introduction

Rabies is a fatal zoonotic disease that continues to be a long-lasting economic burden for developing countries [1]. Globally, rabies is still endemic in over 150 countries and territories causing an estimated 8.6 billion USD worth of economic loss and contributing to over 59,000 human deaths annually (out of which 96% of the cases occur in Asia and Africa) [2,3]. Dogs are the primary reservoir of the rabies virus in Southeast Asia, contributing to 95–99% of the transmission in humans through bites and/or scratches [[4], [5], [6]]. In addition to humans, all warm-blooded domestic animals also contract the rabies virus, for the most part, from a dog bite, and the incidence rate of rabies in animals varies within the species [[7], [8], [9], [10]]. The mucous secretion (e.g. saliva) of an infected animal serves as the predominant source for rabies infection, which is often acquired in both humans and animals through an animal bite and rarely through direct contact of an infected material with an open wound [11,12]. A global call for action to work against rabies unitedly was convened by the World Health Organization (WHO), the Food and Agriculture Organization (FAO), the World Organization for Animal Health (OIE) and the Global Alliance for Rabies Control (GARC) in December 2015. In response, a global strategic framework has endorsed entitled ‘Zero by 30’ to support rabies endemic countries in order to accelerate their national rabies elimination programmes aimed at ending human deaths from dog-mediated rabies by 2030 [13]. This plan has focused on integrating One Health actions with multisectoral engagement for working at human-animal-environmental interface to interrupt in rabies transmission from animals to human [14]. As rabies is an entirely preventable disease, so the One Health efforts by the rabies key stakeholders including health, veterinary, and the local government as well as the extended community people able to cope the disease in efficient way through integrated animal bite management and post-exposure prophylaxis (PEP), scaling of mass dog vaccination (MDV), and community level educational rabies awareness programs [15,16].

Rabies has spread widely in Bangladesh with an annual death incidence of 200 in humans and approximately 25,000 in animals (reported in 2015) [17,18]. There is a large dog-population density (108 dogs/sq.km) and 83% of these dogs are stray dogs with an average annual dog (or other animal) bite incidence of over 0.3 million in humans [18]. In 2010, the Bangladesh government began a rabies elimination programme, which has progressively reduced rabies-related human mortality in recent years [19]. This programme is tackling rabies through prompt action and by providing modern animal-bite management facilities through the establishment of at least one dog-bite management corner in each district hospital, implementing MDV throughout the year and conducting awareness activities [20,21]. All these actions are executed through the One Health approach with the close coordination of human health, animal health, and the local government as well as with the engagement of the community [18]. The success of the One Health initiative relies on enhancing the KAP level among the public and on improving the skill of the personnel directly dealing with animal bite management and suspected rabies patients, including humans and animals. There have been many community-based rabies surveys that have focused only on the community members in Bangladesh and other developing countries [[22], [23], [24], [25], [26], [27], [28]]; however, till date no study in Bangladesh and a minimum number of studies in elsewhere have determined the level of KAP about rabies among other stakeholders of rabies elimination programme [[29], [30], [31], [32]].

Therefore, a baseline study was conducted to understand the extent of KAP about rabies among the frontline human and animal health workers as well as among the community individuals to devise improved intervention strategies and government policies for rabies elimination.

## 2 Materials and methods

This study was conducted under the component of operational research of the Communicable Disease Control (CDC) Unit, Directorate General of Health Services (DGHS), Ministry of Health and Family Welfare (MoHFW), Bangladesh. All the questionnaires and study procedures were reviewed by the institutional technical working group (TWG) members and finally approved by the Director, Disease Control and Line Director, CDC, DGHS, MoHFW, Bangladesh.

### 2.1 Study area

**Fig. 1:**
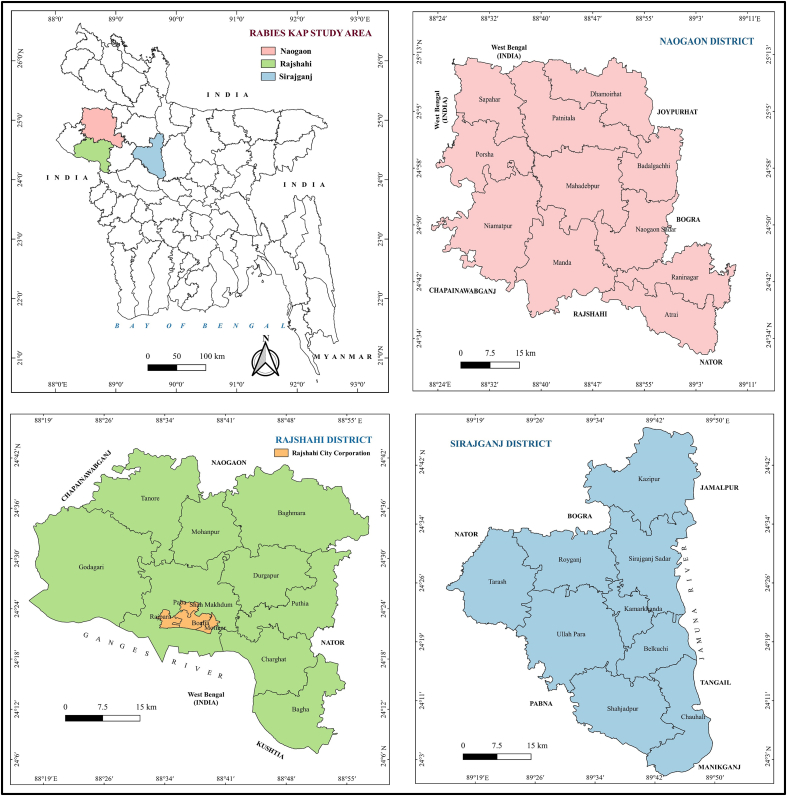
Geographical location of the study areas of rabies KAP survey in Bangladesh, 2016.

A cross-sectional KAP survey on rabies was conducted in the northern territory of Bangladesh between July 2016 and October 2016. Based on the socio-demographic representation, three different districts (Rajshahi, Naogaon and Sirajganj) under the greater administrative division of Rajshahi were selected for this study. Each district is divided into sub-districts, some of which contain city corporations (the most urbanised areas) and/or municipalities (the urban or peri-urbanised areas). According to the 2011 Bangladesh Bureau of Statistics (BBS) census, the geographic location of Rajshahi district lies between 24°07՜ and 24°43՜ Northern latitudes and between 88°17՜ and 88°58՜ Eastern longitudes. Rajshahi has a single city corporation, 14 municipalities and nine sub-districts. The total population of this district is roughly 26,99,688 (1070/sq.km) with an average literacy rate of 53%. The Naogaon district is situated at the Northern side of Rajshahi between the Northern latitudes of 24°32՜ and 25°13՜ and Eastern longitudes of 28°23՜ and 89°10՜. Naogaon consists of 11 sub-districts and three municipalities with a population of roughly 27,01,907 (757/sq.km). The literacy rate is 48%. The Sirajganj district is the gateway of North Bengal and is positioned between 24°01՜ and 24°47’ Northern latitudes and between 89°15՜ and 89°59՜ Eastern longitudes. Sirajganj comprises nine sub-districts, which includes a total of six municipalities with a population of roughly 32,20,814 (1290/sq.km) and a literacy rate 42% (Fig. 1).

### 2.2 Study design and survey questionnaire

This study design employed a cross-sectional perspective. The study was conducted in three different communities in the selected districts: community peoples (CPs), human healthcare professionals (HCPs) and veterinary practitioners (VPs). A structured questionnaire, including closed and open-ended questions (with the local Bengali language translation) was designed to collect data through a face-to-face interview with respondents in respective categories. The study was conducted by 20 veterinary student interns (also the interviewers) from the Department of Veterinary and Animal Sciences at the University of Rajshahi. Prior to the survey, interviewers participated in a day-long orientation workshop to familiarise themselves with the use of the questionnaire, data collection procedures and the consent form held by the trained professionals of the Directorate General of Health Services (DGHS) and the Department of Veterinary and Animal Sciences. Each of the questionnaires considered the socio-demographic profile of the respondents. The questionnaire comprised 25 rabies-related questions regarding rabies knowledge, attitudes towards animal bites and stray dogs, and healthcare-seeking behaviour. Five additional specific questions were included in the questionnaire to assess the professional knowledge level of the HCPs and VPs on handling animal bites and suspected rabies patients and animals (Appendix-A). The respondents were allowed to provide multiple responses (when applicable). The questionnaire was reviewed by the working group members and pretested by a few randomly selected community members to avoid any personal, social or cultural conflict before the actual survey. To be eligible to participate in this survey, an inclusion criterion was set: either the head of the household or an adult member (≥18 years of age) willing and able to provide informed consent. Before the interview, the respondents were briefed about the purpose of the study, and a written or verbal informed consent was obtained. The names of the respondents or other directly identifiable information was not collected as a part of this study. Since no intervention was involved in responding to an interviewer, and all information was kept confidential, participants were not faced any study-related risks.

### 2.3 Community peoples (CPs) survey

**Fig. 2:**
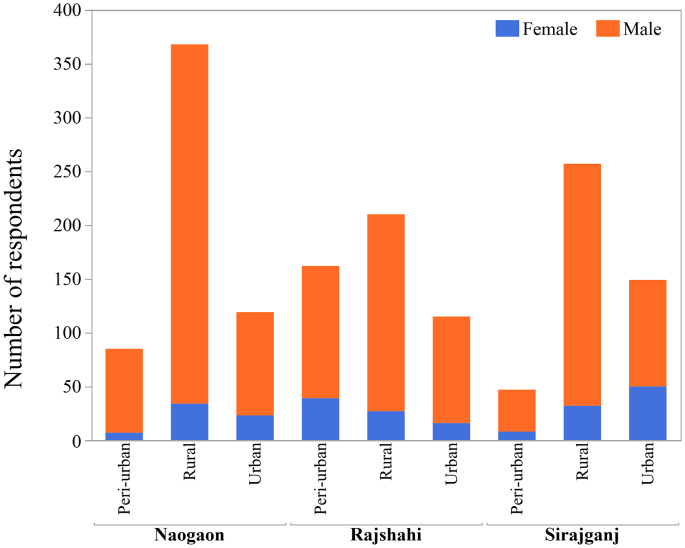
Distribution of respondents of rabies KAP survey in Bangladesh, 2016.

For the CPs survey, we calculated a target sample size of 384 from each district, allowing for a 5% margin of error, a 95% confidence level, 50% expectation with regards to the proportion of people possessing knowledge on rabies (as the proportion of people possessing knowledge about rabies was unknown) and design effect (1.0) using Cochran's sample size formula for categorical data [33]. A cumulative sample size of 1152 was estimated and was spread out among the total number of sub-districts chosen for the study. The target sample size for the interview (number of final respondents) was arrived at by following the systematic random sampling technique (finally arriving at 1133 for the study) (Fig. 2). Interviewers (two-person groups) visited the assigned sub-district on four subsequent occasions in 15-day intervals. They visited the urban or peri-urban sites twice followed by another two visits to the rural sites in respective sub-districts. During each visit, the first household was selected as the central point in areas comprising the city corporation, municipality, union and/or ward (e.g. regional administrative office, zero point, cross street or the largest structure in the visited area). In urban or peri-urban areas, every 25th household was interviewed and in the rural area, every 15th household was interviewed. If the interviewer reached an apartment building, each apartment in the building was counted as a household unit (in the apartments, counting began from the ground floor). If the selected household was empty or if there was no one at home, the next closest household was selected for an interview. When an intersection was reached, a pen was spun to indicate the next direction to be taken. If the interviewer came to the end of an assigned zone, they would backtrack to the central point and the pen would be spun again (which would indicate the new direction of the survey). The heads of the household were first requested for the interview; however, if any single member of the household met the inclusion criteria (in the absence of the head of the household), they were interviewed for this study.

### 2.4 Human healthcare professionals (HCPs) survey

For the KAP about rabies survey among HCPs, we accessed a registered sub-district level hospital and a union health facility and also included some village practitioners (≤ 20% of the sample size) based on their availability by employing a convenience sampling technique in each sub-district. Interviewers (two-person groups) visited the selected health facilities on two consecutive days (in 15-day intervals) and interviewed the healthcare providers who were working in the facility for over six months and willing to participate in the survey. During the second day of the visit, HCPs who participated in the previous day were excluded from the interview and those who met the inclusion criteria were added as new respondents. By the end of this survey, we interviewed a total of 211 HCPs (Fig. 2).

### 2.5 Veterinary practitioners (VPs) survey

For the VPs survey, we selected registered veterinary hospital in the sub-districts and included some para-veterinary volunteers (≤ 20% of the sample size) who usually practised in villages. The selection was done based on their availability by following a convenience sampling technique in each sub-district. Interviewers (two-person groups) visited the selected veterinary hospital facility on two consecutive days (in 15-day intervals) and interviewed animal healthcare providers who were working in the hospital for over six months and willing to participate in the survey. During the second day of the visit, VPs who participated in the previous day were excluded from the interview and those who met the inclusion criteria were added as new respondents. By the end of this survey, we interviewed a total of 168 VPs (Fig. 2).

### 2.6 Data analysis

The data was entered in a Microsoft Excel (2016) spreadsheet (Microsoft Corporation, Redmond, WA, USA), and three individual databases were created with respect to the three studied populations. Subsequently, the individual database was exported into JMP® 14.0 (SAS Institute, Cary, NC, USA) software for descriptive analyses of the respondents' socio-demographic profiles (frequency, proportion and means) and univariate analyses of the explanatory variables, including knowledge on rabies, attitudes and perception responses. A subset of questions was used to determine a respondent's KAP level (Appendix-B). This study selected at least six basic questions focusing on rabies susceptibility, mode of transmission, clinical signs, prevention and control to collectively constitute a minimum indicator of a respondent's satisfactory KAP about rabies. A respondent who did not answer any of the questions correctly was considered as having an unsatisfactory KAP level. Logistic regression analyses were performed to determine the relationship between the respondents' KAP level and their socio-demographic characteristics (with α level set at 0.05).

## 3 Results

### 3.1 Socio-demographic characteristics of the respondents

The socio-demographic profiles of the respondents of the selected populations are presented in Table 1. Out of 1133 CPs, most of the respondents who participated in the survey were from the Naogaon district (39.4%) followed by Rajshahi (31%) and Sirajganj (29.6%) districts. The average age of the respondent was 37 years (range:18–86 years) with the median of 35 years. The majority of the respondents were male (86.1%), lived in rural areas (58.9%) and attended at least secondary school (49.6%). Overall, 25.1% of the respondents were involved with small to large-scale businesses by private firms, 20.6% were farmers, 14.5% were government employee, 12.3% were students and the remaining reported other forms of vocations. The average monthly household income among the surveyed population was 109.87 USD (range: 0–2380.95 USD). One hundred and thirty seven respondents (12.1%) owned household pets, out of which 63 respondents (5.56%) owned dogs and 75 respondents (6.62%) owned cats.

**Table 1:** Socio-demographic profiles of the respondents participating in rabies knowledge, attitudes and perceptions survey in Bangladesh, 2016.

Community people (*N* = 1133)			Healthcare professionals (*N* = 211)			Veterinary practitioners (*N* = 168)		
	n	%		n	%		n	%
District								
Rajshahi	352	31.07	Rajshahi	76	36.02	Rajshahi	60	35.71
Naogaon	446	39.36	Naogaon	67	31.75	Naogaon	61	36.31
Sirajganj	335	29.57	Sirajganj	68	32.23	Sirajganj	47	27.98
Age								
Mean and median of age (range)_ years		36.98, 35, (18–86)	Mean and median of age (range)_ years		37.01, 35, (18–65)	Mean and median of age (range)_ years		43.07, 45, (20–68)
Gender								
Male	975	86.05	Male	136	64.45	Male	165	98.21
Female	158	13.95	Female	75	35.55	Female	3	1.79
Residence								
Rural	667	58.87	Rural	95	45.02	Rural	73	43.45
Urban	226	19.95	Urban	88	41.71	Urban	67	39.88
Peri-urban	238	21.01	Peri-urban	28	13.27	Peri-urban	28	16.67
Education								
No formal education	205	18.09	No formal education	–	–	No formal education	–	–
Primary school	367	32.39	Primary school	–	–	Primary school	–	–
Secondary school	232	20.48	Secondary school	63	29.86	Secondary school	32	19.05
Higher secondary	262	23.12	Higher secondary	109	51.66	Higher secondary	106	63.10
Graduation	68	6.00	Graduation	39	18.48	Graduation	30	17.86
Occupation								
Agriculture	43	3.80	LMFA	11	5.21	Para-vets	18	10.71
Business (private firm)	284	25.07	CHCP	21	9.95	VFA	48	28.57
Day labour	98	8.65	Health Assistant/ Inspectors	28	13.27	Compounder	12	7.14
Farmer	233	20.56	Lab. Assistant/ Technologist	10	4.74	Dresser	16	9.52
Housewife	97	8.56	Pharmacist	6	2.84	AI-technicians	12	7.14
Employed	164	14.47	MT EPI/EPI Technician	3	1.42	FAAI	20	11.90
Professional workers	45	3.97	Senior Staff Nurse/ Nurse	65	30.81	Veterinarian- specialist	13	7.74
Retailers	20	1.77	Interns	14	6.64	Office Assistant	12	7.14
Student	139	12.27	SACMO	10	4.74	Volunteer	17	10.12
Others	10	0.88	Doctor-specialist	15	7.11			
			Volunteer	28	13.27			
Annual Income								
Mean monthly household income, USD (range)		109.87 (0–2380.95)	Mean monthly household income, USD (range)		272.28 (48.81–892.86)	Mean monthly household income, USD (range)		276.15 (23.81–857.14)
Household owing pets								
Yes	137	12.09	Yes	22	10.43	Yes	20	11.90
No	996	87.91	No	189	89.57	No	148	88.10
Household owning dogs	63	5.56	Household owning dogs	10	4.74	Household owning dogs	11	6.55
Household owning cats	74	6.53	Household owning cats	12	5.69	Household owning cats	9	5.36

N·B: CHCP- Community Health Care Provider; LMFA- Local Medical Assistant & Family Welfare; MT EPI- Medical Technologist of Expanded Programme on Immunization; SACMO- Sub Assistant Community Medical Officer; VFA- Veterinary Field Assistant; FAAI- Field Assistant of Artificial Insemination.

Among the 211 HCPs, respondents from Rajshahi, Naogaon and Sirajganj districts contributed to 36%, 31.8% and 32% of the total 211 HCPs respectively. The average age of the respondent was 37 years (range: 18–65 years) with the median age of 35 years. There were more male respondents (64.5%) than females, and a comparable number of respondents resided in rural (45%) and urban (41.7%) areas. At least half (51.7%) of the total number of participants had completed higher secondary education (Class XII) and 18.5% had completed graduation (honour's degree); whereas, 29.8% of the HCPs had a secondary education (Class VI-X) background. In this study, doctors comprised 7.1% of the total number of respondents, interns contributed to 6.64%, sub-assistant community medical officers (SACMO) contributed to 4.74%, nurses contributed to 30.8%, community health care providers (CHCP) contributed to 9.95% and health inspectors and assistants contributed to 13.27%. Individuals working in the aforementioned fields were involved with patient care and management. The respondents who were indirectly involved with healthcare facilities and village practitioners were 9% and 18.48% respectively. The average household income of the respondents among the HCPs was 272.28 USD (range: 48.81–892.86 USD). A total of 22 respondents (10.43%) owned household pets, out of which 10 respondents (4.74%) owned dogs and 12 respondents (5.69%) owned cats.

The respondents interviewed among the VPs were 36.3% in Naogaon district, followed by Rajshahi 35.7% and Sirajganj 28%. The mean and median age of the respondents was 43.1 years (range: 20–68 years) and 45 years respectively. There were more males (98.2%) than females, and most of the respondents who participated were from rural areas (43.45%) followed by urban (39.9%) and peri-urban (16.67%) areas. Sixty-three percent of the VPs had completed higher secondary education, 17.8% completed graduation and the remaining 19.1% appeared for secondary school. The majority (28.6%) of the respondents were veterinary field assistants (VFA), 11.9% of the respondents were field assistants of artificial insemination (FAAI), 9.5% were dressers, both compounders and artificial insemination (AI) technicians were counted as 7.14%; whereas, 7.74% of the total number of respondents were veterinarians and 20.8% served as para-veterinarians and volunteers. The average monthly household income among the VPs was 276.15 USD (range: 23.81–857.14 USD). Approximately 12% (*n* = 20) of the respondents owned pet animals, out of which 11 respondents (6.55%) owned dogs and 9 respondents (5.36%) owned cats.

### 3.2 Knowledge on rabies transmission and diagnosis

A substantial knowledge gap existed among the CPs about rabies transmission and diagnosis (Table 2). The majority (84.73%) of the CPs were familiar with the fact that an animal bite might cause a disease; however, only 49.43% of the CPs provided the name of the disease correctly: rabies. The respondents from all populations understood that, predominantly, humans could be infected by the rabies virus; however, a high percentage of the respondents (particularly among the CPs) did not possess sufficient knowledge that domestic, pet and wild animals could also be infected with rabies. Out of 1133 CPs, 377 respondents (33.27%) were unaware about the final consequences of the disease resulting from animal exposure. Over 95% of the respondents believed that dogs were primarily responsible for causing rabies. At the same time, 34.33% of the CPs, 51.66% of the HCPs and 52.38% of the VPs believed that cats could cause rabies. A small percentage of the respondents had insufficient knowledge about the fact that wild animals could also transmit the rabies virus to humans. Approximately 50% of the respondents among both HCPs and VPs and 30% among CPs assumed that it was unlikely for the rabies virus to transmit through scratches in addition to bites. CPs relied on social media as their main source (34.8%) of knowledge about rabies and other diseases followed by books, television and newspapers. Most of the participants' opinions on the diagnosis of rabies was that the disease could be confirmed by a laboratory test.

**Table 2:** Respondents knowledge on rabies transmission and diagnosis surveyed by rabies KAP study in Bangladesh, 2016.

Rabies knowledge	Community people (N = 1133)		Healthcare professionals (N = 211)		Veterinary practitioners (N = 168)	
	n	%	n	%	n	%
Do you know any disease caused by an animal's bite?						
Yes	960	84.73	207	98.10	164	97.62
No	173	15.27	4	1.90	4	2.38
Answered correctly the name of the disease as rabies						
Yes	560	49.43	207	98.10	164	97.62
Who can be infected in rabies?						
Human	1001	88.35	202	95.73	128	76.19
Cattle-Goat-Sheep	584	51.54	114	54.03	102	60.71
Dog	166	14.65	57	27.01	55	32.74
Cat	95	8.38	35	16.59	39	23.21
Mongoose	8	0.71	–	–	–	–
Others	–	–	–	–	2	1.19
What is the fate of rabies?						
100% fatal	756	66.73	187	88.63	153	91.07
Not fatal	172	15.18	18	8.53	5	2.98
Cured automatically	23	2.03	–	–	3	1.79
Don't know	178	15.71	4	1.90	4	2.38
Which animal is responsible for rabies?						
Dog	1118	98.68	207	98.10	161	95.83
Cat	389	34.33	109	51.66	88	52.38
Fox	184	16.24	82	38.86	63	37.50
Jackal	80	7.06	44	20.85	34	20.24
Mongoose	44	3.88	21	9.95	23	13.69
Monkey	18	1.59	13	6.16	12	7.14
Others	12	1.06				
How is rabies transmitted?						
Bite	1053	92.94	204	96.68	166	98.81
Scratch	340	30.01	104	49.29	86	51.19
Licking	25	2.21	7	3.32	11	6.55
Touching	9	0.79	–	–	4	2.38
How do you know about disease including rabies?						
Television	206	18.18	15	7.11	12	7.14
Social program	394	34.77	62	29.38	68	40.48
Newspaper	53	4.68	11	5.21	5	2.98
Books	230	20.30	133	63.03	102	60.71
Do you believe that rabies can be confirmed by laboratory test?						
Yes	751	66.28	152	72.04	128	76.19
No	382	33.72	59	27.96	39	23.21

### 3.3 Attitudes towards animal bite management and rabies

The findings from the study about the attitudes of the respondents towards animal bites and rabies are presented in Table 3. The table indicates that there is clearly a negative attitude among the CPs about washing wounds with soap and water (28.68%), and a positive attitude of healthcare-seeking behaviour resulting from medical care (49.43%) compared to traditional healers (16.15%). A significant number of HCPs (35%) and VPs (40%) were also found to believe medical consultation is unnecessary in response to animal bites. Upon enquiring about where the rabies vaccine could be found, 76%, 88% and 73% of the respondents among CPs, HCPs and VPs respectively answered the District Rabies Prevention and Control Centre (DRPCC) that is the district level referral centre for dog bite cases and suspected rabies patients. All the bite incidence occurring at the tertiary level are referred by the sub-district level hospital or community-based clinic to the DRPCC for modern bite management and post-exposure prophylaxis. Over 90% of the respondents from all categories possessed a negative attitude towards dogs, as they believed that stray dogs are a major source for rabies in Bangladesh; however, 24% among CPs, 30% among HCPs and 37% among VPs believed that dogs are their friends.

**Table 3:** Respondents attitudes towards animal bite management and rabies surveyed by rabies KAP study in Bangladesh, 2016.

Rabies attitudes	Community people (N = 1133)		Healthcare professionals (N = 211)		Veterinary practitioners (N = 168)	
	n	%	n	%	n	%
What measures do you think should be taken following an animal exposure?						
Wash with water	56	4.94	23	10.90	12	7.14
Wash with soap and water	325	28.68	162	76.78	139	82.74
Consult with traditional healers	183	16.15	–	–	–	–
Consult with local doctors	158	13.95	7	3.32	3	1.79
Consult with physicians and receive vaccine	560	49.43	137	64.93	101	60.12
Nothing to do	13	1.15	–	–	–	–
Where is the rabies vaccine found in your area?						
DRPCC	856	75.55	185	87.68	123	73.21
Municipality	176	15.53	25	11.85	30	17.86
Pharmacy	231	20.39	22	10.43	37	22.02
Is a stray dog a major problem for causing rabies in Bangladesh?						
Yes	1054	93.03	194	91.94	157	93.45
No	77	6.80	17	8.06	9	5.36
Dogs are our friend, what do you think?						
Agree	273	24.10	64	30.33	62	36.90
Disagree	860	75.90	147	69.67	106	63.10

### 3.4 Perceptions about rabies prevention and control

Perceptions on rabies prevention and control are indicated in Table 4. The study found that 20% of CPs had been exposed to animal bites, including dog bites at least once in their lifetime. The percentage of animal bite exposure among HCPs and VPs was 18% and 14% respectively. The perception level about the prevention of rabies by a vaccine among the participants was high (82% among CPs, 98% among HCPs, and 98% among VPs). The majority of the CPs (62%) believed that killing stray dogs was the best way to prevent and control rabies, while a higher percentage among HCPs (67%) and VPs (70%) believed that mass dog vaccination (MDV) could be an efficient tool to neutralise the rabies virus in dogs and thus, prevent it from spreading further to humans and other animals. On average, 93% of the respondents from all populations were convinced that the dog population needs to be controlled in Bangladesh, but their perceptions on population management strategies varied. The majority of the CPs (67%) provided their opinion on culling dogs. On the other hand, 45% of the respondents, among both HCPs and VPs, suggested that sterilisation could be an appropriate control measure. The study reported that 137 (12%) of the CPs, 22 (10%) among HCPs and 20 (12%) among VPs owned pets. A poor perception level with regards to the recommended annual vaccination of pets was observed among all groups (12%, 14% and 20% respectively). A beneficial practice, that of disposing dead animals was observed among the participants (98% and above in all groups).

**Table 4:** Respondents perceptions about rabies prevention and control surveyed by rabies KAP study in Bangladesh, 2016.

Rabies perceptions	Community people (N = 1133)		Healthcare professionals (N = 211)		Veterinary practitioners (N = 168)	
	n	%	n	%	n	%
Have you ever been bitten by animal?						
Yes	223	19.68	38	18.01	23	13.69
No	910	80.32	173	81.99	145	86.31
Do you believe that rabies can be prevented by a vaccine?						
Yes	933	82.35	206	97.63	164	97.62
No	197	17.39	5	2.37	4	2.38
What do you think to control rabies in Bangladesh?						
Mass dog vaccination	437	38.57	142	67.30	117	69.64
Killing of stray dogs	702	61.96	65	30.81	49	29.17
Animal birth control	41	3.62	11	5.21	10	5.95
Do you have pets in your house?						
Yes	137	12.09	22	10.43	20	11.90
No	996	87.91	189	89.57	148	88.10
Have you ever vaccinated your pets for rabies?						
Yes	50	36.50	14	63.64	9	45.0
No	87	63.50	8	36.36	11	55.0
How often should a pet be vaccinated against rabies?						
Once vaccinated	30	21.90	10	45.45	5	25.0
Annually vaccinated	17	12.41	3	13.64	4	20.0
Non-vaccinated	90	24.32	9	4.91	11	55.0
Is it important to control the dog population in Bangladesh?						
Yes	1067	94.17	198	93.84	155	92.26
No	64	5.65	13	6.16	12	7.14
What methods is/are appropriate to control dog population in Bangladesh?						
Sterilisation	327	28.86	94	44.55	75	44.64
Impounding	15	1.32	8	3.79	7	4.17
Sterilisation and impounding	43	3.80	24	11.37	23	13.69
Culling	757	66.81	95	45.02	66	39.29
What do you do when an animal die?						
Burying in the ground	1074	94.79	189	89.57	150	89.29
Floating in the pond or river	7	0.62	2	0.95	3	1.79
Burn	57	5.03	21	9.95	14	8.33

### 3.5 HCPs and VPs knowledge and practices on animal bite management

As per the data indicated in Table 5, 76% of the respondents among HCPs had sufficient knowledge on the WHO category of animal bites; however, 12% of the respondents did not possess knowledge on how to manage an animal bite properly. Although 93% of the HCPs understood the clinical signs and symptoms presented by rabies, close to 35% of the HCPs had never observed a patient afflicted with rabies. Over 88% of the participants suggested the animal bite victims for taking post-exposure prophylaxis rather than visiting traditional healers (1.42%) or local practitioners to consider medical treatment (10.43%).

**Table 5:** Practices of human healthcare professionals and veterinary practitioners for treating animal bite wounds surveyed by rabies KAP study in Bangladesh, 2016.

Specific questions to human healthcare professionals and their responses	n	%
Do you know about the category of animal bites?		
Yes	162	76.78
No	49	23.22
Have you ever seen a rabies patients?		
Yes	138	65.40
No	73	34.60
Do you know how to properly manage an animal bite case?		
Yes	185	87.68
No	26	12.32
Do you know about the clinical signs and symptoms of human rabies?		
Yes	197	93.36
No	12	5.69
What do you suggest if someone has been bitten by animals?		
Refer for vaccination	186	88.15
Refer for traditional healers	3	1.42
Suggest for medicinal treatment	22	10.43

Specific questions to veterinary practitioners and their responses		
Have you ever seen rabid animals?		
Yes	152	90.48
No	16	9.52
Have you ever treated any suspected rabid animals?		
Yes	87	51.79
No	81	48.21
Have you ever taken pre-prophylaxis for rabies?		
Yes	48	28.57
No	120	71.43
Do you know about the clinical signs and symptoms of animal rabies?		
Yes	155	92.26
No	12	7.14
What you suggest if you see an animal that is infected with rabies?		
Suggest treatment	45	26.79
Culling of the infected animals	123	73.21

The majority (92%) of the VPs were aware about the clinical signs and symptoms presented by rabid animals, and a higher percentage (90%) of the VPs had seen rabid animals at some point of time in their respective fields. However, a concerning factor was that 52% of the VPs had experience in treating rabid animals at least once in their professional careers of which 72% of the respondents had no history of taking pre-exposure prophylaxis. Moreover, among the respondents, 27% of the VPs suggested entrusting the responsibility of the treatment of rabid animals to their owners instead of using precaution and culling a rabid animal themselves.

### 3.6 Factors associated with the level of KAP about rabies

The respondents' KAP level about rabies and its related factors are presented in Table 6. Among people in the community, a higher percentage (56%) of the respondents from the Rajshahi district were found to have a satisfactory level of KAP about rabies; this level was three times higher than that of Sirajganj (30%) and Naogaon (21%) districts. There was a significant relationship between the lack of formal education (or respondents who completed only primary schooling) and poor KAP about rabies (*P* ≤ 0.01). Additionally, the odds of having a satisfactory KAP level was 1.5 times greater for those with a higher secondary education than with those who had completed graduation. The findings from the result revealed that the KAP level among the male respondents was 23% lower than the female respondents. Urban people had a 38% lower KAP than the peri-urban population, but a 53% higher level of KAP than people with rural subsistence. With HCPs, a higher percentage of the respondents (63%) in the Rajshahi district had a satisfactory level of KAP when compared with the Sirajganj district (34%) and the Naogaon (43%) district. The odds of having a satisfactory KAP about rabies among the HCPs in Rajshahi was 1.71 times higher than in Sirajganj. The study found that the level of KAP increased with the level of education (*P* ≤ 0.01). More female respondents (19%) possessed satisfactory KAP than males. However, KAP among the respondents who resided in urban communities was 54% higher than in rural communities and 37% higher than in peri-urban communities. Among the VPs, the level of satisfactory KAP about rabies was reported as 70%, 42% and 26% in Rajshahi, Sirajganj and Naogaon districts respectively. The respondents in the Rajshahi district had a 1.58 times higher KAP level than in Sirajganj. The level of education was directly proportional to an increase in the KAP level among the VPs. The study showed 48% higher KAP among the female respondents than in males. However, the extent of satisfactory KAP about rabies was 19% and 18% higher among the VPs who resided in urban constituencies than among VPs who resided in rural and peri-urban constituencies respectively.

**Table 6:** Factors associated with the level of knowledge, attitudes and perceptions about rabies among different group of people surveyed by rabies KAP study in Bangladesh, 2016.

Variable	Category	Community people				Human healthcare professionals				Veterinary practitioners			
		Satisfactory	Unsatisfactory	OR (95%)	*P*-value	Satisfactory	Unsatisfactory	OR (95%)	*P*-value	Satisfactory	Unsatisfactory	OR (95%)	*P*-value
District	Rajshahi	198 (56.25)	154 (43.75)	3.02 (2.21, 4.14)	<0.001	48 (63.16)	28 (36.84)	1.71 (0.88, 3.34)	0.11	42 (70.00)	18 (30.00)	1.58 (0.71, 3.53)	0.26
	Naogaon	95 (21.30)	351 (78.70)	0.64 (0.46, 0.88)	0.01	29 (43.28)	38 (56.72)	0.76 (0.39, 1.50)	0.43	16 (26.23)	45 (73.77)	0.24 (0.11, 0.55)	<0.001
	Sirajganj	100 (29.85)	235 (70.15)	1.00		34 (50.00)	34 (50.000	1.00		28 (41.79)	19 (58.21)	1.00	
Education level	No formal education	47 (23.74)	158 (76.26)	0.63 90.36, 1.10)	0.002	–	–	–		–	–	–	–
	Primary school	98 (26.70)	269 (73.30)	0.49 (0.29, 0.84)	0.01	–	–	–		–	–	–	–
	Secondary school	81 (34.91)	151 (65.09)	0.72 (0.42, 1.25)	0.25	25 (39.68)	38 (60.32)	0.20 (0.08, 0.49)	<0.001	14 (43.75)	18 (56.25)	0.68 (0.25, 1.85)	0.45
	Higher secondary	138 (52.67)	124 (47.33)	1.50 (0.87, 2.56)	0.14	56 (51.38)	53 (48.62)	0.32 (0.14, 0.73)	0.01	52 (49.06)	54 (50.94)	0.84 (0.37, 1.90)	0.68
	Graduation	29 (42.65)	39 (57.35)	1.00		30 (76.92)	9 (23.08)	1.00		16 (53.33)	14 (46.67)	1.00	
Gender	Male	330 (33.85)	645 (66.15)	0.77 (0.55, 1.09)	0.14	69 (50.74)	67 (49.26)	0.81 (0.46, 1.43)	0.46	84 (50.91)	81 (49.09)	0.52 (0.05, 5.83)	0.59
	Female	63 (39.87)	95 (60.13)	1.00		42 (56.00)	33 (44.00)	1.00		2 (66.67)	1 (33.33)	1.00	
Residence	Rural	175 (26.24)	492 (73.76)	0.47 (0.35, 0.65)	0.09	34 (35.79)	61 (64.12)	0.46 (0.26, 0.84)	0.01	31 (42.47)	42 (57.53)	0.81 (0.41, 1.57)	0.53
	Peri-urban	121 (50.84)	117 (49.16)	1.38 (0.95, 1.98)	<0.001	12 (42.86)	16 (57.14)	0.63 (0.26, 1.49)	0.28	12 (42.86)	16 (57.14)	0.82 (0.34, 2.00)	0.66
	Urban	97 (42.92)	129 (57.08)	1.00		48 (54.55)	40 (45.46)	1.00		32 (47.76)	35 (52.24)	1.00	

## 4 Discussion

The “United against rabies” collaboration through the One Health approach is the current motto for rabies elimination strategy globally. This collaboration is the first ever of its kind in Bangladesh that covers the evaluation of KAP about rabies among the members of rabies key stakeholders as a part of the One Health approach contributing to the rabies elimination programme. The timing of the study was unique, as it was accomplished at least half a decade after the national rabies programme launched. The study also began just prior to the start of the global one health initiative against rabies. The findings addressed here would serve as an indicator to strengthen the existing policies and actions as well as provide a baseline for the future assessment of the national programme towards rabies elimination in Bangladesh and other countries with a similar socio-economic status by 2030.

In this study, half of the CPs were not able to correctly provide the name of the disease, despite possessing a fair amount of knowledge on rabies. The findings correspond to a study conducted by Alam et al. [20] who reported that 58% of people were unaware about the rabies, while over one-third of the respondents among the CPs were unaware about the consequences of the disease. The findings were consistent with another study conducted in Ethiopia (67.8%) [22], but the results were higher than the findings obtained from a study conducted in Morocco (10.2%) [34].

Dogs are mainly responsible for causing rabies. This is well-reported and known worldwide, including in Bangladesh [19,[35], [36], [37], [38]]. This study found that over 95% of the respondents from all populations possessed a high awareness about the transmission of rabies from dogs; however, there was a substantial gap regarding rabies transmission dynamics through domestic and wild animals. Similar results were found in other developing countries [24,29,[39], [40], [41]]. Moreover, the majority of the CPs and over half of both the HCPs and the VPs possessed insufficient knowledge about the nature of exposure that leads to rabies. The reason for this gap was because the respondents were unaware that humans and/or animals could be exposed to the rabies virus by a scratch in addition to bites. Digafe et al. [22] observed a similar knowledge gap among the community people in Ethiopia. This revealed that the primary source for respondents in the CPs to gain awareness about rabies was social media. In addition, similar to other studies [21,28], television, books and newspapers also contributed to the dissemination of information about rabies.

From this study, we found that the attitudes among the CPs towards animal bite management were not very positive, as only 29% of the respondents knew that the wound should be washed immediately with soap and water. About 49% of the respondents showed a positive attitude to seek treatment from physicians and receive post-exposure prophylaxis (PEP). The percentage of the respondents who were unaware about wound washing was higher than the findings obtained from a previous study in Bangladesh (2%) [21]. The findings were consistent with studies conducted in countries, such as India (31%) and Ethiopia (30.7%) [42]. However, we found a stark difference in the improvement of attitudes towards healthcare-seeking behaviour from traditional healers in other studies conducted in Bangladesh [21,28]. This might have occurred due to an improvement in the socio-demographic status of the population and an increasing number of awareness campaigns in the areas where the present study was conducted. Moreover, the households included in this study were located in four distinct points of each sub-district (around the centre of the areas where the study was conducted). The results may have been different around the areas where marginalised communities live. We noticed that a significant percentage of HCPs (35%) and VPs (40%) bore a negative attitude towards the treatment of wounds in government health facilities but were fine with seeking treatment elsewhere. This could also be a reason for poor accessibility of the anti-rabies vaccine in government health facilities, as the demand for the vaccine in the government supply chain throughout the country is remarkably high.

The findings from our study revealed that the respondents had a negative attitude towards stray dogs, as they believed that a high density of stray dogs was the main obstacle for rabies prevention and control and that culling could be the best choice to control the dog population. The findings were consistent with another study conducted by Bouaddi et al. [34] who reported that 45% of the respondents in the survey did not agree to dog sterilisation and 54.3% provided their opinion in favour of culling. Regular vaccination of dogs and well-planned sterilisation methods could reduce rabies transmission and dog population in the community [19,43]. Respondents among HCPs and VPs had the aforementioned perception. This might be due to their professional engagement with dog bite incidence management and animal exposure. In contrast, pet owners did not employ adequate practises for the vaccination of their pets. These findings were inconsistent with Ntampaka et al. [30] study in Rwanda who reported that 58% owners had their pets vaccinated at least once a year. This might be due to socio-economic variation with Bangladesh and indicates lack of best practices that need to be improved through awareness campaigns.

In this study, the respondents among the HCPs possessed sufficient knowledge in animal bite management. Despite this, 23% of the HCPs were not adequately trained on the category of animal bites, which is much lower than the findings obtained by a study conducted in Haiti (85%) [29]. Therefore, despite possessing sufficient knowledge, we recommend that HCPs need to attend continuous refresher trainings to ensure proper wound management and learn about appropriate usage of vaccines. This study revealed that over 51% of the VPs had experience in rabid animal treatment. However, only 29% of the VPs had previously taken pre-exposure prophylaxis. These findings should be of great concern to veterinary practitioners because of a high risk of being exposed to the rabies virus during the handling of rabid animals. The livestock officials need to enact measures to ensure that VPs take pre-exposure prophylaxis to minimise the risk of rabies transmission.

There was a strong relationship between poor levels of KAP about rabies and a lack of formal education among the community members. These results are in agreement with findings in other studies, which have found that an increased level of education relates to a higher level of knowledge on rabies [24,25,44,45]. Additionally, the residential status also had an effect on the level of KAP about rabies. The respondents in urban and peri-urban populations had more awareness on rabies than the people living in rural areas, which corresponds with the study conducted by Alam et al. [20] who concluded that a sufficient knowledge about rabies highly correlated with people living in urban areas because of the education facilities and an increased standard of living.

The dynamics of rabies transmission is solely animal originated that can be prevented by vaccinating the reservoir animals, particularly dogs. Inadequate knowledge of dog vaccination, limited vaccinating resources at community level and a greater number of stray dogs remain a large proportion of dogs unvaccinated and human at risk for rabies exposure [46]. Vaccinating at least 70% dogs in three consecutive years through MDV could attain a significant reduction in the virus transmission [47,48]. Regarding health-seeking behaviours of dog bite victims, recent studies showed that rabies victims sought treatment from by traditional healers (66.8%) instead of visiting hospital and receiving PEP which is the reflection of lack of formal education on rabies at the community level in Bangladesh [19,38]. Through increasing community level education, knowledge about dog bites and its consequences, and especially necessity of taking PEP measures to reduce the risk of becoming rabies infection can be improved. Moreover, proper management of animal bites cases in both human and veterinary sector with trained staffs and the accessibility of rabies vaccine at government facilities are necessary to reduce human rabies death cases [38]. It would be preferable to arrange refresher training on time basis for both human and veterinary personnel's at tertiary and district level hospitals to improve their work efficiency and motivation. A joint efforts of the respective departments by activating One Health strategy could be anticipated these challenges in the best possible way [15,16]. Bangladesh has recently started to give rabies as a national priority, and has taken actions to address the problems through One Health approaches with strong political commitments and budget allocation that is a positive milestone towards rabies elimination [18]. However, the sustainability of the working enforcement and funding is necessary to achieve the goal ‘zero’ dog-mediated rabies by 2030. The findings of this study are promising and need to be incorporated in current national strategies and policies to address the remaining obstacles on the basis of real practical collaboration and practices.

The study has some limitations. The most important is the representation of the study population in HCP and VP groups, where participation of the respondents was based on the convenience sampling methods and a considerable number of respondents came from health care providers and veterinary assistants, respectively, due to the shortage of manpower at the tertiary level hospitals in both sectors. Meanwhile, we did not performed KAP analyses according to their roles that is the weakness in our study. In addition, few other circumstances may have an influence on respondent's responses such as lack of understanding of particular questions due to the low level of education status of most of CP participants, recent activities like MDV conducted in some study areas, and less number of female respondent participated under the VP group.

## 5 Conclusion

There is still scope to increase KAP about rabies among the people in the community, particularly those living in rural areas. Mass awareness programmes and dissemination of rabies-related information through mass media to the communities could be useful to bridge this gap. Healthcare providers and veterinary service providers need proper training and education programmes to ensure proper animal bite management. They should also be provided with the knowledge on how to use vaccines effectively on both humans and animals. Finally, the integrated One Health approach through the participation of all main stakeholders of rabies elimination program with sustainable commitments at both national and international levels and providing resources is critical to achieve the goal of rabies elimination by 2030.

## Conflict of interests

The author(s) have no competing interests to declare.

## Supplementary data

Appendix A: Rabies KAP survey questionnaire Image 1

Appendix B: Respondent's rabies KAP calculation chart Image 2
