# Supplementary material for: Knowledge, attitudes and perceptions about rabies among the people in the community, healthcare professionals and veterinary practitioners in Bangladesh
Source: One Health. 2021 Aug 14;13:100308. doi: 10.1016/j.onehlt.2021.100308 (PMC8379336; doi:10.1016/j.onehlt.2021.100308)
Supplement: Supplementary file 1 — Appendix A: Rabies KAP survey questionnaire [file mmc1.docx]

| **Group of Study:**   - Community people - Healthcare professionals - Veterinary practitioners |
| --- |

| Communicable Disease Control (CDC) of DGHS and Dept. of Veterinary and Animal Science of Rajshahi University is conducting a comprehensive surveillance on KAP Study on Rabies among a group of people. We are selected you as a respondent of this group. Are you agree to give your opinion: (Remember that your answers will be confidential)  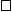 Yes 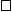 No |
| --- |

| **Knowledge, Attitude and Practice (KAP) Study on Rabies** | | | | | |
| --- | --- | --- | --- | --- | --- |
| **Sl. No.** | | | | | **Interview date:** |
| Name of the Respondent (Adult): | |  | | | Mobile: |
| Respondent Details: | | Father’s/ Husband’s name: | | | |
|  |  | Age (yrs.): | | | Sex: Male Female |
|  |  | Occupation: | | | Monthly income: |
|  |  | Religious: Muslim/Hindu/ Buddhist/ Christian | | | Family size: 1-3/ 4-6/ 7- Above |
|  |  | Education: No formal education Primary Secondary Higher secondary University | | | |
| Address: | | Village/Para/Rd # | | | Ward # |
|  |  | Union: | | | Post Office: |
|  |  | Upazila/ Thana: | | | District: |
|  |  | Residence: Rural Urban Pari/Sub- Urban | | | |
| **Q.** | **Please answare the following questions** | | | | |
| 1. | Have you ever been bitten by animal? | | Yes No | | |
|  |  |  | If yes-... Dog Cat Fox Jackal Mongoose Monkey Others:……… | | |
|  |  |  | Type of animal: Stray Community Own Pet Others:…………......... | | |
| 2. | Do you know any disease caused by animal bite/ scratch? | | Yes No | | |
|  |  |  | If yes …..please specify name of the disease:……………………………………..... | | |
| 3. | Have you heard about rabies? | | Yes No | | |
| 4. | Who can be infected in rabies? | | Human Cattle-Goat-Sheep Dog Cat Mongoose Others: …………………………... | | |
| 5. | What is the fate of rabies? | | 100% Fatal disease Not Fatal Cured Automatically Not known | | |
| 6. | Which animal is responsible for rabies? | | Dog Cat Fox Jackal Mongoose Monkey Others:………………. | | |
| 7. | How is rabies transmitted? | | Animal bite Animal scratch Animal licking Touching Others: ………... | | |
| 8. | How can rabies be prevented? | | Vaccine Traditional treatment (kabiraj) Traditional Treatment (Magician) | | |
| 9. | Do you believe that rabies can be prevented by vaccine? | | Yes No | | |
| 10. | How do you know it? | | Tevevision Social program Newspaper Reading book Others: ……... | | |
| 11. | Where is rabies vaccine found? | | District Sadar Hospital Municipality Pharmacy | | |
| 12. | What measures do you take following an animal bite? | | Wash with water  Wash with soap and water  Consult with kabiraj  Consult with local doctors  Consult with physicians and receive vaccine  Nothing to do  Others:…………………………………………………………………………….. | | |
| 13. | What treatment you seek if someone bitten by an animal? | | Hospital Traditional healer (kabiraj) Traditional healer (Magician)  Nothing to do | | |
| 14. | What do you think to control rabies in Bangladesh? | | Mass Dog Vaccination Killing of stray dogs Animal Birth Control (ABC)  Others: …………………… | | |
| 15. | Do you have pets in the house? | | Yes No | | |
|  |  |  | If yes-... Dog Cat Mongoose Others:…… | | |
| 16. | Have you ever been vaccinated your pets for rabies? | | Yes No | | |
|  |  |  | Once-vaccinated Annually vaccinated Non-vaccinated Unknown | | |
|  |  |  | If No …Reseason: Lack of Knowledge Lack of Attention Cost of Vaccine | | |
| 17. | What do you do when you see your animal is seek? | | | Provide treatment Killed Nothing | |
| 18. | Is stray dog a problem in Bangladesh | | | Yes No | |
| 19. | Is it important to control dog population in Bangladesh? | | | Yes No | |
| 20. | Do you believe that rabies can be prevented by Mass Dog Vaccination (MDV)? | | | Yes No | |
| 21. | What methods is/are appropriate to control dog population in Bangladesh? | | | Sterilization  Impounding  Sterilization and impounding  Kiliing | |
| 22. | Do you believe that rabies can be confirmed by laboratory test? | | | Yes No | |
| 23. | Do you believe that there are no locally available treatments for rabies? | | | Yes No | |
| 24. | Do you know about the disposal of dead animals? | | | Yes No  If yes, Specify?  Filling in the ground  Floating in the ponds/river  Burning  Nothing have to do | |
| 25. | Dog is our friend or not, what you think? | | | Yes No | |
| **Specific questions for the human healthcare professionals** | | | | | |
| 1. | Do you know about the category of animal bite?  How many bite category according to WHO? | | | Yes No | |
|  |  |  |  | Please, specify…………………………………………. | |
| 2. | Have you ever seen rabies patient? | | | Yes No | |
| 3. | Do you know about proper animal bite management? | | | Yes No | |
| 4. | Do you know about clinical sign and symptoms of human rabies? | | | Yes No If yes, please specify…. | |
|  |  |  |  | Hydrophobia Photophobia Aerophobia | |
| 5. | What you suggestion if someone bitten by animal? | | | Reffered for vaccinatin  Reffered for traditional treatment  Provide medicinal treatment | |
| **Specific questions for the veterinary professionals** | | | | | |
| 1. | Have you ever seen rabid animals? | | | Yes No | |
| 2. | Have you ever treated rabid animals? | | | Yes No | |
| 3. | Have you ever been taken pre-prophylaxis of anti-rabies vaccine? | | | Yes No | |
|  |  |  |  | If No …Reseason:  Lack of Knowledge  Lack of Attention  Cost of Vaccine | |
| 4 | Do you know about clinical symptoms of rabid animals? | | | Yes No If yes, please specify…. | |
|  |  |  |  | Anorexia Aggression Salivation Lethargy | |
| 5. | What you suggest if you see a rabies-infected animal? | | | Provide medicinal treatment  Killing of Euthenized of the animal  Selling in the market | |

| **Thanks for your active participation.** | Signature:  **Interviewer:** ……………………………………….….  Date: ………………………………………….............. |
| --- | --- |
