# Supplementary material for: Knowledge, attitudes and perceptions about rabies among the people in the community, healthcare professionals and veterinary practitioners in Bangladesh
Source: One Health. 2021 Aug 14;13:100308. doi: 10.1016/j.onehlt.2021.100308 (PMC8379336; doi:10.1016/j.onehlt.2021.100308)
Supplement: Supplementary file 2 — Appendix B: Respondent's rabies KAP calculation chart [file mmc2.docx]

**KAP CALCULATION CHART**

- **KAP calculations of the community people**

| **Questions** | **KAP calculations of the community people** | |
| --- | --- | --- |
|  | **Satisfactory** | **Unsatisfactory** |
| 1. Do you know any disease caused by animals bite/scratch? | Yes  Correctly write the name of the disease | No  Yes, but did not write correctly the name of the disease |
| 1. Who can be infected in rabies? | Human  Cattle-Goat-Sheep  Dog  Cat | Did not answered any of them |
| 1. What about the fate of rabies? | 100 % fatal | Not fatal  Cured after treatment or automatically  Not known |
| 1. Which animal is responsible for human rabies? | Dog  Cat  Fox  Jackal  Mongoose  Monkey | Did not answered dog and cat |
| 1. Where is the rabies vaccine found? | District Sadar Hospital | City-corporation or Municipality  Pharmacy |
| 1. What measures do you take following animal bite? | Wash wound with soap and water  Consult with physicians and receive vaccine | Wash with water  Consult with kabiraj  Consult with local doctors  Nothing to do |

| **Questions** | **KAP calculations of the healthcare professionals** | |
| --- | --- | --- |
|  | **Satisfactory** | **Unsatisfactory** |
| 1. Do you know any disease caused by animals bite/scratch? | Yes  Correctly write the name of the disease | No  Yes, but did not write correctly the name of the disease |
| 1. Who can be infected in rabies? | Human  Cattle-Goat-Sheep  Dog  Cat | Did not answered any of them |
| 1. What about the fate of rabies? | 100 % fatal | Not fatal  Cured after treatment or automatically  Not know |
| 1. Which animal is responsible for human rabies? | Dog  Cat  Fox  Jackal  Mongoose  Monkey | Did not answered dog and cat |
| 1. Where is the rabies vaccine found? | District Sadar Hospital | City-corporation or Municipality  Pharmacy |
| 1. What measures do you take following animal bite? | Wash wound with soap and water  Consult with physicians and receive vaccine | Wash with water  Consult with kabiraj  Consult with local doctors  Nothing to do |
| 1. Do you know category of animal bite? And how many bite according to WHO? | Yes  3 types and explained clearly | No  Yes, but did not write 3 types and explained clearly |
| 1. Do you know clinical signs of human rabies? | Yes  Hydrophobia, photophobia and aerophobia | No  Yes, but did not answered any of them |

- **KAP calculations of the healthcare professionals**
- **KAP calculations of the veterinary practitioners**

| **Questions** | **KAP calculations of the veterinary practitioners** | |
| --- | --- | --- |
|  | **Satisfactory** | **Unsatisfactory** |
| 1. Do you know any disease caused by animals bite/scratch? | Answered yes and correctly write the name of the disease | Answered no, or yes but did not write correctly the name of the disease |
| 1. Who can be infected in rabies? | Human  Cattle-Goat-Sheep  Dog  Cat | Did not answered any of them |
| 1. What about the fate of rabies? | 100 % fatal | Not fatal  Cured after treatment or automatically  Not known |
| 1. Which animal is responsible for human rabies? | Dog  Cat  Fox  Jackal  Mongoose  Monkey | Did not answered dog and cat |
| 1. Where is the rabies vaccine found? | District Sadar Hospital | City-corporation or Municipality  Pharmacy |
| 1. What measures do you take following animal bite? | Wash wound with soap and water  Consult with physicians and receive vaccine | Wash with water  Consult with kabiraj  Consult with local doctors  Nothing to do |
| 1. Do you know clinical signs of animal rabies? | Yes  Anorexia, Aggression, salivation, lethargy | No  Yes, but did not answered any two them |
